# Correlates of Non-Medical Prescription Drug Misuse Among a Treatment-Seeking Population: A Comparison with Illicit Drug Users

**DOI:** 10.3390/ijerph15091978

**Published:** 2018-09-11

**Authors:** Asharani PV, Edimansyah Abdin, Tan Jun Wen, Mythily Subramaniam, Christopher Cheok, Guo Song

**Affiliations:** 1 Research Division, Institute of Mental Health, 10 Buangkok View, Singapore 539747, Singapore; edimansyah_abdin@imh.com.sg (E.A.); mythily@imh.com.sg (M.S.); 2 National Addictions Management Service, Institute of Mental Health, 10 Buangkok View, Singapore 539747, Singapore; junwen.tan2@gmail.com (T.J.W.); song_guo@imh.com.sg (G.S.); 3 Forensic Psychiatry, Institute of Mental Health, 10 Buangkok View, Singapore 539747, Singapore; Chris_CHEOK@imh.com.sg

**Keywords:** prescription drugs, illegal drugs, drug abuse, addiction severity, quality of life, correlates

## Abstract

Prescription drugs (PD) undoubtedly help people with various physical or psychiatric ailments. Nevertheless, they are often diverted and misused (use without prescription or for purposes/in ways not intended by the prescriber). This study compared the sociodemographic and clinical correlates of those who misused PDs, used illegal drugs and co-ingested both, to identify those who were at a high risk of misusing these drugs. Retrospective analysis of the treatment outcome monitoring (TOM) data for the period of 2013–2017 identified 1369 subjects for the study; 295 patients presented with PD use disorder (PDUD alone), 811 with illegal drug use disorder (IDUD alone), and 263 had both PDUD and IDUD. The study sample included treatment seeking population (Singaporeans and permanent residents). TOM data included data collected through direct interviews (addiction severity, quality of life) and from the clinical case notes (diagnosis, co-morbidities, socio demographic information, etc.). The most commonly misused prescription and illegal drugs were benzodiazepines (63.1%) and heroin (63.4%), respectively. Those who co-ingested both PD and illegal drugs (PDUD+IDUD) had a significantly higher addiction severity score, lower quality of life and higher psychiatric co-morbidities than that of IDUD alone at baseline. When compared to Chinese patients, Malay and Indian patients had lower odds (*p* < 0.05) of PDUD alone and PDUD+IDUD than Chinese patients; divorcees had higher odds of PDUD+IDUD than those who were married. Those with primary and secondary qualifications had higher odds (2.1 and 2.9 times, respectively) of PDUD+IDUD than those with tertiary qualification and those in managerial or professional roles had higher odds of PDUD alone than those who were unemployed. Gender, ethnicity, marital status, education and occupational classes were associated with PDUD and IDUD. These characteristics can be helpful to identify those who are at the risk of PDUD and incorporate strict prescription monitoring to their care.

## 1. Introduction

Drug use disorder has affected around 0.6% of adults globally, claiming 17 million years of healthy life [1]. The perils of substance use are perceptible as the rising number of drug overdoses in the U.S. Among these deaths, 66% overdoses involved prescription or illicit opioids [2]. The diversion of prescription medications has emerged as a drug epidemic in the past few decades. The National Epidemiologic Survey on Alcohol and Related Conditions (NESARC) in the U.S. showed a lifetime prevalence of 4.7%, 4.1% and 3.4% for opioid, sedative and tranquilizer misuse, respectively [3]. In the U.S., during 1992–2002, prescription drug (PD) dispensing increased by 154.3%. This included an increase in prescriptions for opioids by 222%, benzodiazepines by 49.2% and stimulants by 368.5% [4].

Like illicit drug use, PD misuse (use of PD without prescription or in a manner or dose unintended by the prescribing clinician, or the use of PDs to experience euphoria or using someone else’s medication even for legitimate reasons (e.g., pain treatment) [5,6]) has serious psychosocial and economic impact that includes higher rates of psychiatric comorbidities, suicidality and unemployment [7,8,9,10]. A National Survey on Drug Use and Health reported that 15.1 million people in the US misuse PDs which is 50 times higher than heroin [4,11]. Several studies have highlighted that the individuals misusing PDs are likely to develop an addiction for other licit/illicit drugs [3,12,13]. Mortality and morbidity are the major health challenges among those with non-medical prescription drug misuse and illicit drug use. In the US alone, a 2.8-fold surge in prescription drug- related overdose deaths were documented from 2001 to 2014. Prescription opioids and benzodiazepines contributed to a 3.4 and 5 fold rise in overdose deaths, respectively, during the same period [14]. Apart from the mortality due to drug overdose, opioid misuse has pervasive health and social/economic ramifications. White et al. [15] reported that inpatient admissions among those with opioid use disorder were 12 times higher than non-users, leading to an 8-fold increase in mean annual health costs for the same group. In 2011, over half (51%) of the emergency department visits were contributed by illicit drugs whilst 1.2 million visits involved PDs [16].

Not many studies have been conducted in Asia to understand the prevalence of PD misuse and its consequences. Surveys conducted in Asian countries indicated misuse of opioid based PD in many South Asian countries [17,18,19,20], PDUD accounted for 12.1% of addictions in India [19]. Singapore has strict drug laws enforced under the “Misuse of Drugs Act” that carry tough penalties including execution, if convicted. The substances covered under the law include both illegal and prescription drugs. Offences involving PDs (e.g., nimetazapam) also face similar legal consequences as illegal drugs. A study conducted locally among those with chronic non-cancer pain has indicated methadone (45.2%), morphine (38.1%), oxycodone (23.8%) and fentanyl (9.5%) being the most prescribed pain medications [21]. Prescription opioids such as buprenorphine are highly discouraged in Singapore following a rapid increase in buprenorphine-related deaths in 2006 [22]. The stricter policies, differing availability of prescription drugs and differences in prescription practices are unique to Singapore that make the comparison with international data difficult. There is also a paucity of data on the PD misuse situation in Singapore and the differences in the characteristics of those who misuse PDs or use illegal drugs. Therefore, this study is timely and important in Singapore both from a policy as well as clinical perspective. National Addictions Management Service (NAMS) is the main national provider of addiction services in Singapore. The treatment outcome monitoring programme (TOM) at NAMS follows the patients at regular intervals to capture their treatment outcome. The data captured as part of the TOM were analysed retrospectively for the period of 2013–2017 to analyse the prescription drug misuse situation in Singapore. Characteristics of the patients, type of PD or illegal drugs abused were studied to understand the correlates of substance use that could be used to identify those at a higher risk of misusing PDs and illegal drugs and to develop targeted treatment strategies for the group. To reflect the recent changes adopted by the DSM-5, a neutral term “use disorder” has been adopted in this manuscript. The term PDUD includes misuse, abuse and dependence without differentiating the degree of severity. Likewise, the term IDUD includes all types of illegal drug use (abuse and dependence) without differentiating the severity.

## 2. Materials and Methods

### 2.1. Participants

A total of 2273 treatment seeking individuals from Singapore (Singaporeans and permanent residents) were identified from the 2013 to 2017 data collection period, from those who consented for the study. TOM excludes forensic cases, those who explicitly deny any addiction related issues, cases mandated by the ministries/courts, patients who are aggressive suicidal and do not have the mental capacity to understand the questions. Those who were misusing PDs alone (PDUD, misused drugs that were prescribed for various medical conditions, e.g., valium, codeine, tramadol, etc.), using illegal drugs alone (IDUD, included heroin, cannabis, cocaine, methamphetamines, etc.) or co-ingested both PD and illegal drugs (PDUD+IDUD) were included in the analysis. A detailed list of commonly available prescription and illegal drugs in Singapore are included in Appendix A. The data represents PDs that are being misused and doesn’t include those which are prescribed for existing medical conditions. The distinction was made through self-report of misuse, frequency of misuse and clinicians assessments. Subjects who used alcohol alone (pure alcohol users) were excluded. The variables used in the analysis were drawn from the TOM databases which are routinely used in the analysis of treatment outcomes. These included variables collected through the interview (drug type, frequency, severity etc.) and from the clinical case notes (e.g., psychiatric co-morbidities, drug types, diagnosis, etc.). The study adhered to the Declaration of Helsinki and the procedures approved by the Domain Specific Review Board of the National Healthcare Group, Singapore (Ref: 2010/00373). All the subjects included in the study gave informed consent for inclusion.

### 2.2. Measurements

TOM employs several standardised measures to track the treatment outcome of the patients which includes the severity of specific addictions and the personal well-being of the patients, in addition to clinically relevant information such as socio-demographic factors, medical, psychiatric history, and addiction-related behaviours which are extracted from the clinical case notes. Addiction severity and quality of life measures were tracked at the baseline and 3 months follow up to monitor the treatment outcome while the rest of the variables (both sociodemographic and clinical variables) were captured during their first visit to the clinic.

#### 2.2.1. Socio Demographic Information (Gender, Ethnicity, Education, Occupation and Marital Status)

The socio demographic information collected included gender (male/female), ethnicity (Chinese/Malay/Indian/others), marital status (single/married/divorced/widowed), education (no formal education/primary/secondary/tertiary (pre-university and university)), occupation (clerical/labourer/manager, etc.). These were captured from the electronic medical records which were verified from the identification documents or captured through self-report. Clinical information was collected from the electronic medical records.

#### 2.2.2. Diagnosis of Substance Use Disorder

The diagnosis was captured from the electronic medical records and denotes the diagnosis given during the first visit (ICD-10) for their substance use.

#### 2.2.3. Co-Morbidity

Psychiatric co-morbidities were captured by the clinicians using the standard clinical procedures and included only active diagnosis (mood disorder, adjustment disorder, insomnia, psychosis, anxiety disorder, conduct disorder and dementia). This refers to the diagnosis made by the doctors either at the intake session (first visit) or during the referral. Self-reported diagnosis was not captured for this variable. Physical comorbidities (blood borne diseases, diabetes, hyperlipidemia, endocrine disorders, liver diseases, asthma, conditions related to gastrointestinal, urinary and respiratory tracts, autoimmune conditions, cancer and cardiovascular diseases) on the other hand, were largely self-reported, except for the blood borne diseases which are confirmed by the doctors through the blood tests.

#### 2.2.4. Severity of Substance Use: Addiction Severity Index-Lite (ASI-Lite)

The type of substances misused were captured through ASI-Lite (self-report) and verified through urine drug tests (if patient consents) and against the medical records. The ASI-Lite is a semi-structured scale that assesses patients in the following domains: medical, employment and support, substance use, legal problems, family and social matters, and psychiatric issues [23]. Relevant information is captured across the lifetime, as well as the previous 30 days. The ASI-Lite is administered to patients who declare addictions to substances such as alcohol and drugs. Composite scores (responses divided by the maximum value for that response and calculating the sum of every responses in that domain) were calculated from problem areas which were used in the analysis. Medical use of PDs (prescribed by doctors for medical conditions) was not captured by the ASI.

#### 2.2.5. Quality of Life: Personal Well-Being Index (PWI)

The PWI is an 8-item measure of subjective well-being measured for the past 1 month period. Satisfaction with the domains—standard of living, overall health, achievements in life, personal relationships, personal safety, community connectedness, and future security—was measured on an 11-point Likert scale ranging from 0 to 10, indicating complete dissatisfaction to complete satisfaction respectively. Composite scores are calculated by converting each item to a percentage of scale maximum, achieved by shifting the decimal point to the right (e.g., a score of 7 converts to 70% SM), and then averaging the sum of total percentile scores. Composite scores range from 0 to 100, wherein higher scores demonstrate higher levels of personal well-being [24]. The scale has exhibited high levels of validity in cross-cultural studies [25], especially so for populations with Chinese majorities [26].

### 2.3. Procedure

Patients seeking treatment at the NAMS clinic meet the TOM coordinator prior to their appointment with the doctor or the counsellor. During this interview, the interviewer administers the aforementioned assessments (ASI-Lite and PWI) most relevant to their present addictions if the patient is agreeable to participate. Those who participate in the TOM during the baseline visit are re-administered the TOM at their 3rd and 6th month follow up if they are still returning for treatment. The rest of the variables were captured from the clinical case notes from the first visit.

### 2.4. Analyses

Data analysis was performed using STATA version 13.1 (Stata Corp., Texas, TX, USA). Descriptive statistics were first conducted to examine the frequency and percentage for categorical variables and mean and standard deviation for continuous variables. To determine how demographical traits affect the odds of PDUD, and PDUD+IDUD with IDUD as a reference, a multinomial logistic regression analysis was performed. The model considered the following variables—gender (i.e., male/female), ethnicity (i.e., Chinese/Malay/Indian) marital status at baseline (i.e., single/married), and highest educational qualification attained (i.e., primary/secondary). The selection of sociodemographic correlates was guided by the previous literatures [3,9,27] and significant associations (*p* < 0.05) in the univariate analyses using Chi square test (for categorical variables) and independent sample *t*-test (for continuous variables). Repeated measures ANOVA test was run to identify any significant differences in composite drug scores between pre and post treatment for patients who used PDs or illegal drugs exclusively, and patients who used both exclusively. One-way ANOVA was also run to identify any significant differences in composite scores at baseline between three groups. All statistical significant difference was set at *p* value < 0.05.

## 3. Results

### 3.1. Demographics

Of the 2273 subjects identified for the study, 904 used alcohol exclusively (no drugs) and were excluded from the analysis. Of the remainder, 295 had PDUD alone, 811 had IDUD alone, and 263 had PDUD+IDUD. Those with PDUD alone were predominantly male (Table 1, 82.4%, *n* = 243), Chinese (71.3%, *n* = 164), either single or married (39.5%, *n* = 111; 39.5%, *n* = 111), and unemployed (54.8%, *n* = 153). Those with IDUD alone were primarily male (89.6%, *n* = 727), either Chinese (41.3%, *n* = 277) or Malay (39.6%, *n* = 266), single (53%, *n* = 412), and unemployed (51.7%, *n* = 399). Subjects with PDUD+IDUD were primarily male (89%, *n* = 234), Chinese (60.4%, *n* = 128), single (43.1%, *n* = 106), and unemployed (57.4%, *n* = 144). The mean age of those with PDUD alone was 41.2 (SD = 11.4), IDUD alone was 41 (*n* = 811, SD = 14.0), and those with PDUD+IDUD was 45.2 (*n* = 263, SD = 11.2).

Of the 295 subjects with PDUD alone, one could not identify the drug they considered their ‘main drug’, defined as the substance sought out and used the most. Of the remaining 294 with PDUD alone, 63.1% (*n* = 186) used benzodiazepines as their main drug, and 18.6% (*n* = 55) used codeine. For the subjects with IDUD alone (*n* = 811), 63.4% (*n* = 514) used heroin as their main substance, 18.7% (*n* = 152) used methamphetamines and 9.1% (*n* = 74) cannabis. For the subjects with PDUD+IDUD (*n* = 263), 61.6% (*n* = 162) used heroin as their main drug, 14.1% (*n* = 37) used benzodiazepines, and 11.8% (*n* = 31) used amphetamines.

### 3.2. Severity of Addictions

The mean baseline severity scores (ASI-Lite) for those with PDUD alone, IDUD alone and PDUD+IDUD was 0.2 (SD = 0.1), 0.2 (SD = 0.1), and 0.3 (SD = 0.2), respectively. Those with PDUD+IDUD had a significantly higher ASI-Lite scores than those with PDUD alone (Mean difference = 0.1, *p* < 0.001) and IDUD alone (Mean difference = 0.05, *p* < 0.001) at baseline. Significant improvement in scores (decreased severity) was found within the group for those with PDUD alone; *t* (65) = −5.2, *p* < 0.0001, IDUD; *t* (139) = −7.7 *p*<.001, and PDUD+IDUD; *t* (39) = −2.9, at *p* = 0.007 at 3 months follow up. Nevertheless, the improvement was not significantly different between the three groups, F (2) = 0.04, *p* = 0.958.

### 3.3. Quality of Life

The mean baseline PWI scores for those with PDUD alone was 48.5 (SD = 18.6), IDUD was 50.3 (SD = 21.3), and for those with PDUD+IDUD was 46.5 (SD = 20.8). One-way ANOVA revealed that those with PDUD+IDUD had a significantly lower scores than that of IDUD alone at baseline (Mean difference = 3.8, *p* = 0.04). At the 3 month review, 22.4% (*n* = 66) of those with PDUD alone returned for their follow up consultations, compared to 17.3% (*n* = 140) of those with IDUD alone, and 15.2% (*n* = 40) of patients with PDUD+IDUD. Returning subjects reported a mean increase of PWI scores (better quality of life) of 59.2 (SD = 17.5) for PDUD alone, 58.4 (SD = 21.0) for IDUD alone, and 57.2 (SD = 21.5) for PDUD+IDUD (Table 2).

While no significant differences were found between the pre and post treatment PWI scores across the three groups, significant improvements were found for those with PDUD alone; *t* (65) = −4.7, *p* < 0.001, and IDUD alone; *t* (139) = −2.9, *p* = 0.005.

### 3.4. Co-Morbidities: Psychiatric and Physical

The prevalence of psychiatric comorbidity (48.1% (PDUD alone), 35.7% (PDUD+IDUD) and 19.4% (IDUD alone), *p* < 0.01) and anxiety disorder (6.8% (PDUD alone), 2.3% (PDUD+IDUD) and 0.7% (IDUD alone), *p* < 0.01) were significantly higher in PDUD alone and in PDUD+IDUD than in IDUD alone. Mood disorders (20% (PDUD and 10.2% (IDUD), *p* < 0.01) and adjustment disorders (10.5% (PDUD alone) and 5.3% (IDUD alone), *p* < 0.01) were significantly more prevalent in those with PDUD alone than in IDUD alone. Those with IDUD alone had significantly higher physical co-morbidities (*p* < 0.001) than those with PDUD alone and PDUD+IDUD. Among physical co-morbidities, respiratory tract related conditions formed the largest group (24.0%).

### 3.5. Predictors of Drug Use

Compared to Chinese patients, Malay and Indian patients had lower odds (0.3 (*p* < 0.001)) of PDUD alone than IDUD alone (Table 3). Marital status was associated with PDUD alone with singles evidencing 0.4 times (*p* < 0.001) lower odds of PDUD alone than married patients. Occupational classes were associated with PDUD with those working as manager/administrator or professional had times increased odds of PDUD alone (4.4 (*p* = 0.047) and 7.4 (*p* = 0.02)) than unemployed.

For patients with PDUD+IDUD, one-year increase in age was associated with 1.03 higher odds of misusing these drugs. Malay and Indian patients had lower odds (0.6 (*p* = 0.015) and 0.4 (*p* = 0.003)) of such co-ingestion when compared to Chinese patients. Divorcees had increased odds (1.8 (*p* = 0.025)) of PDUD+IDUD compared to married patients. Patients with primary and secondary qualifications had 2.1 (*p* = 0.047) and 2.9 (*p* = 0.002) times higher odds of PDUD+IDUD compared to those with tertiary qualifications. Patients with technical or vocational occupations had higher odds (2.4 (*p* = 0.013)) of PDUD+IDUD while those in service or sales industry had lower odds (0.3 (*p* = 0.048)) of PDUD+IDUD than patients who were unemployed. No significant associations were found for gender, ethnicities that did not fall within the others subset, patients who were single, separated but were not yet divorced, widows and widowers, patients with no formal educations, and patients not with technical or vocational occupations.

## 4. Discussion

Predictors of different classes of drugs, such as PDs and illegal drugs are an important research topic considering the soaring global rates of overdoses. Amidst the outcry to curb drug overdoses, a comparison of various factors that predict licit or illicit drug misuse/use is warranted. We compared the sociodemographic and clinical correlates of those who misused PD, used illegal drugs and co-ingested both. Our results showed that benzodiazepine (63.1%) followed by codeine (18.6%) were the most prevalent prescription drugs among the treatment seeking group whilst heroin and methamphetamine were preferred by the IDUD alone group. We did observe a significant difference in the severity of drug use at baseline between the three groups. The group with PDUD+IDUD had a significantly higher severity score and lower QoL than the other two groups. The severity of drug use reduced significantly over the follow up period for all three groups. This suggests that licit/illicit drugs impart its negative effects in users, regardless of the substance type, however this can be minimised with proper intervention. In Singapore, the treatment relies mainly on psychotherapy as substitution therapy is not legalised. Therefore, an improvement in severity scores and other outcomes are clinically encouraging.

We observed a higher prevalence of psychiatric co-morbidities in those with PDUD+IDUD and PDUD alone. This is in line with previous observations where a correlation was observed between substance use and psychiatric conditions [28,29]. Presence of comorbidities can make the treatment process challenging. Unless treated simultaneously with the addiction, it will result in poorer treatment outcomes. We do not know if the co-morbidities are secondary to the PDUD. We also observed a higher prevalence of the mood disorder and adjustment disorder in those with PDUD alone than in other groups which are supported by findings from previous research studies who reported a similar trend [3]. Perhaps a targeted treatment for specific co-morbidities could abate the severity of the diseases.

Ethnicity is recognised as a factor that predicts the type and amount of drugs used by subjects [30,31]. We observed that compared to the other study groups, those of Malay and Indian ethnicity were more likely to use illegal drugs than Chinese. Singapore is a multi-ethnic country. Hence, these differences in drug use patterns among different ethnicities can help in the planning of awareness programmes. Similarly, marriage is suggestive to be protective against illegal drug use and the majority of the subjects in all 3 categories were single. A strong family bond has been identified as a protective factor against drug use in multiple international studies [32]. A stable marriage has been shown to result in better treatment outcomes in subjects who were drug users/misusers [33] whilst unmarried subjects suffered exacerbation of their symptoms. Raskin et al. [34] showed that being in a stable marriage promoted abstinence in cocaine users. It is conceivable that the unwavering support of the spouse or family members acts as the driving force for this change.

We also observed that occupation and lower education status were associated with drug use. Patients with lower education status (primary and secondary qualifications) had higher odds of co-ingesting both drugs (than illegal drugs alone) compared to those with tertiary qualifications. Johnson et al. [35] observed that gaining a higher education is likely to reduce illicit drug use and PD misuse among adolescents. Therefore, higher education is likely to be a protective factor against complicated drug use involving multiple drugs. Similarly, distinct patterns were observed for employment status and roles. Those who were unemployed were less likely to have PDUD+IDUD compared to those with technical or vocational jobs. Those who occupy professional and managerial roles were 4.3 times and 7.3 times, respectively, more likely to have PDUD alone than IDUD alone when compared to those who were unemployed, whilst those in technical and sales jobs were more likely to have PDUD+IDUD than IDUD alone. Studies have shown that unemployment is a risk factor for substance use [36] and risky drug use behaviours [37]. Supporting the literature, we observed significant differences in PD misuse and illegal drug use patterns among those who were employed and unemployed. Those who were unemployed or occupying sales or technical jobs were more likely to use illegal drugs, whereas those occupying higher job roles tend to misuse PDs than illegal when compared to unemployed subjects.

Unemployment among drug users could be attributed to the stigmatisation attached to drug use, unwillingness or inability to work [38]. Re-engaging them back to the labour market and promoting addiction treatment could be viable options to reduce their illicit drug use. We observed that approximately half of the subjects in our study were employed/unemployed. A National Survey on Drug Use and Health (NSDUH) conducted in the US reported that about half (55.1%) of the adults with the substance use disorder were employed full time [39] which supports our observation (Table 1). Finding from a national survey conducted by Perlmutter and Colleagues [40] indicated that the correlates of prescription drug use with job status vary within the different classes of prescription drugs; those who are unemployed had higher odds of using prescription opioid whilst non-full time employment showed higher odds of using prescriptions stimulants. The role of the type of occupational classes in PD misuse is thus of utmost importance to understand the mode of access to these drugs. With this knowledge in hand, deterrence interventions can be made more effective to tailor the characteristics of each group.

Several limitations apply to the current study. The study represents the treatment seeking population alone who are mostly older adults; hence, adolescents are not represented in the study. Also, the age of onset of drug use was not captured in the database we used for analysis which limited the comparison of that variable across the groups. Self-reported nature of the data capture is another limitation of the datasets. Additionally, the addiction clinic has a low treatment adherence with approximately 70% of patients fail to return for their 3 months follow up. Treatment dissatisfaction, incarceration, etc. are presumed to be some of the reasons for this low turn up rate.

## 5. Conclusions

In summary, this study identified key differences in the characteristics of those who misuse PD, use illegal drugs and co-ingest both drugs. We observed differing associations between ethnicity and PDUD. Marriage and education were protective against illicit drug use and those who were employed were more likely to use drugs, with those in higher job rank using prescription drugs. This is an important observation which needs to be explored further to identify the factors that contribute to this discrepancy. Awareness efforts should be channeled towards this group to minimize the diversion of prescription drugs. At present, most of the community awareness programmes are directed towards youngsters to prevent early drug initiation. Our results show the need to expand the efforts to the adults especially individuals with different education levels occupying various job grades. Education was associated with complicated drug use (PDUD+IDUD) with those with lower education tend to involve in co-ingestion of PDs and illegal drugs. The severity scores were significantly different among those who co-ingested both PDs and illegal drugs together than the pure users. The QoL was significantly lower in this group (PDUD+IDUD) which didn’t improve over the time, unlike in the other groups. The treatment outcomes for this group are unlikely to be favorable based on these finding. Those who present with both PDUD+ DUD must be given attention to continuously track their progress and specialized therapies must be implemented to ensure that the severity of addiction and QoL improves with treatment. Based on the identified characteristics clinicians can focus on specific treatment programs or strict prescription monitoring for those who are at a higher risk of misusing the prescription drugs. While strict prescription monitoring is practiced for certain drugs (e.g., buprenorphine) other PDs with abusive potential slip through the safety net of regular monitoring. Attention must be given to improve the prescription drug monitoring programme to minimize the negative effects of PD misuse.

## Figures and Tables

**Table 1:** Socio-demographic and clinical characteristics of the subjects.

	PDUD Alone *n* (%)	IDUD Alone *n* (%)	PDUD+IDUD *n* (%)
**Gender**			
Male	243 (82.4)	727 (89.6)	234 (89)
Female	52 (17.6)	84 (10.4)	29 (11)
**Ethnicity**			
Chinese	164 (71.3)	277 (41.3)	128 (60.4)
Malay	42 (18.2)	266 (39.6)	61 (28.8)
Indian	16 (7)	95 (14.2)	17 (8)
Others	8 (3.5)	33 (4.9)	6 (2.8)
**Marital status**			
Single	111 (39.5)	412 (53)	106 (43.1)
Married	111 (39.5)	226 (29)	70 (28.5)
Separated	6 (2)	16 (2.1)	7 (2.8)
Divorced	50 (17.8)	119 (15.3)	62 (25.2)
Widowed	3 (1.1)	5 (0.6)	1 (0.41)
**Education**			
No Formal Education	3 (1.4)	5 (0.8)	2 (1)
Primary	57 (27.5)	214 (33.5)	74 (36.8)
Secondary	96 (46.4)	297 (46.6)	112 (55.7)
* Tertiary	51 (24.6)	122 (19)	13 (6.5)
**Occupation**			
Clerical/Secretary	2 (0.7)	2 (0.3)	2 (0.8)
Labourer	20 (7.2)	67 (8.7)	18 (7.2)
Manager/Administrator	9 (3.2)	5 (0.6)	2 (0.8)
Others	60 (21.5)	211 (27.3)	54 (21.5)
Professional	9 (3.2)	2 (0.3)	1 (0.4)
Services/Sales	17 (6.1)	51 (6.6)	6 (2.4)
Technical/Vocational	9 (3.2)	35 (4.5)	24 (9.6)
Unemployed	153 (54.8)	399 (51.7)	144 (57.4)
**Comorbidities**			
Psychiatric comorbidity	142 (48.1)	157 (19.4)	94 (35.7)
Anxiety disorder	20 (6.8)	6 (0.7)	6 (2.3)
Mood disorder	59 (20)	83 (10.2)	38 (14.5)
Adjustment disorder	31 (10.5)	43 (5.3)	17 (6.5)
Physical co-morbidity	63 (21.4)	237 (29.2)	65 (24.7)
	**Mean (SD)**	**Mean (SD)**	**Mean (SD)**
Personal wellbeing index	48.52 (18.58)	50.27 (21.27)	46.53 (20.76)
Addiction Severity index	0.19 (0.13)	0.21 (0.13)	0.26 (0.15)

* Tertiary education refers to all pre-university (junior college, technical educations, etc.) and university educations.

**Table 2:** Severity and QoL: The severity and QoL scores at baseline and 3 months follow up.

	Baseline Mean (SD)			3 Months Mean (SD)			Repeated Measure ANOVA Test	
	PDUD Alone	IDUD Alone	PDUD+IDUD	PDUD Alone	IDUD Alone	PDUD+IDUD	F (df)	*p*
**PWI**	48.5 (18.6)	50.3 (21.3)	46.5 (20.8)	59.2 (17.5)	58.4 (21.0)	57.2 (21.5)	0.95 (2)	0.39
**ASI**	0.19 (0.1)	0.21 (0.1)	0.25 (0.2)	0.07 (0.1)	0.06 (0.1)	0.08 (0.1)	0.04 (2)	0.96

**Table 3:** Predictors of drug use: Socio-demographic characteristics that predicts PD or illicit drug use.

	PDUD+IDUD vs. IDUD Alone			PDUD Alone vs. IDUD Alone		
Characteristic (Referent)	OR	*p*	95% CI	OR	*p*	95% CI
Age	1.03	0.003 *	(1.0,1.0)	1	0.201	(1.0, 1.0)
Gender Male vs. Female	1.2	0.651	(0.6, 2.1)	1.4	0.208	(0.8, 2.4)
Ethnicity (Chinese)						
Malay	0.6	0.015 *	(0.4, 0.9)	0.3	0.000 *	(0. 2, 0.4)
Indian	0.4	0.003 *	(0.2, 0.7)	0.3	0.000 *	(0.2, 0.5)
Others	0.4	0.058	(0.2, 1.0)	0.4	0.026 *	(0.2, 0.9)
Marital Status (Married)						
Single	1.2	0.481	(0.8, 1.8)	0.4	0.000 *	(0.3, 0.7)
Separated	1.5	0.496	(0.5, 4.6)	0.8	0.676	(0.3, 2.4)
Divorced	1.8	0.025 *	(1.1, 2.9)	0.8	0.360	(0.5, 1.3)
Widowed	1.4	0.794	(0.1, 15.1)	1. 5	0.713	(0.2, 10.6)
Education (Tertiary)						
No Formal Education	0.7	0.788	(0.1, 7.6)	0.8	0.793	(0.2, 4.2)
Primary	2.1	0.047 *	(1.0, 4.3)	0.6	0.106	(0.4, 1.1)
Secondary	2.9	0.002 *	(1.5, 5.7)	0.8	0.411	(0.5, 1.3)
Occupation (Unemployed)						
Manager/Administrator	2.5	0.347	(0.4, 16.5)	4.4	0.047 *	(1.0, 18.6)
Professional	2.4	0.485	(0.2, 28.2)	7.4	0.020 *	(1.4, 39.4)
Technical/Vocational	2.4	0.013 *	(1.2, 4.6)	0.7	0.454	(0.3, 1.7)
Services/Sales	0.3	0.048 *	(0.1, 1)	0.8	0.539	(0.3, 1.8)
Labourer	0.6	0.097	(0.3, 1.1)	0.9	0.723	(0. 5, 1.7)
Others	0.8	0.205	(0.5, 1.2)	0.7	0.114	(0. 5, 1.1)

* Statistically significant.

## Appendix A

**Table A1:** The common prescription and illegal drugs available in Singapore used in the analysis.

Illegal Drugs	Prescription Drugs
Methamphetamine (Ice, Yaba, etc.) Heroin Cannabis Cocaine Ecstasy New Psychoactive Substances (NPS, synthetic cannabinoids, synthetic cathinone, etc.) *	**CNS depressants** *Barbiturates* Phenobarbital *Benzodiazepines* Alprazolam Lorazepam Diazepam Clonazepam Nitrazepam Medazolam *Z-hypnotics* Zopiclone Zolpidem Amitriptyline *Antihistamines* Chlorpheniramine Promethazine Diphenhydramine
	**Stimulants** Methylphenidate Modafinil Amphetamine
	**Centrally acting appetite suppressant** Phentermine
	**Anticholinergics** Benzhexol Benztropine
	**Opioid agonist** Codeine Tramadol Morphine Oxycodone Hydromorphone Meperidine Methadone Buprenorphine
	**Over the counter Preparations** *Decongestants* Ephedrine Pseudoephedrine *Anti-diarrhea* Loperamide Diphenoxylate *Cough preparations* Containing Codeine, Dextromethophan
	**Others** Ketamine Steroids Propofol

The drugs in the italics denotes the main drug classes; * Source: Central Narcotics Bureau, Singapore (Drug report 2017). The list of prescription drugs has been compiled from the NAMS pharmacy drug list.
